# Metastatic Hepatoblastoma in Adolescence: A Clinical Case Report and Literature Review

**DOI:** 10.7759/cureus.58460

**Published:** 2024-04-17

**Authors:** Juan Manuel Millan-Alanis, Luis A González Torres, Alfonso Ferrant-Noo, Joel Isai Alcala-Gonzalez

**Affiliations:** 1 Department of Internal Medicine, Hospital Universitario Dr. Jose Eleuterio Gonzalez, Universidad Autonoma De Nuevo Leon, Monterrey, MEX

**Keywords:** liver and gall bladder disease, : adolescence, gastrointestinal oncology, hepatoblastoma, case report

## Abstract

Hepatoblastoma is the most common hepatic neoplasm in children. However, its incidence is infrequent beyond age five. We present the case of a 15-year-old female diagnosed with metastatic hepatoblastoma during hospitalization for liver function deterioration. The patient presented with abdominal distension, jaundice, and other symptoms indicative of advanced disease. Imaging and biopsy confirmed stage IV epithelial hepatoblastoma with pulmonary metastases. This case underscores the importance of considering hepatoblastoma in older pediatric patients or young adults presenting with hepatic masses despite lacking traditional risk factors for liver malignancies.

## Introduction

Hepatoblastoma is a rare tumor that presents almost exclusively in children; it is the most common primary hepatic neoplasm, and more than 90% of cases occur before the age of two. Most cases are sporadic, and their incidence decreases with age. Adult and adolescent case reports are rare [1-3]. We present the case of a 15-year-old female diagnosed with metastatic hepatoblastoma during hospitalization for a diagnostic workup of worsening liver function. Few articles have reported adolescent cases of advanced hepatoblastoma tumors, and case reporting represents an important task.

## Case presentation

A 15-year-old female of Hispanic origin presented to the hospital with increased abdominal circumference and shortness of breath; she denied any relevant family or medical history. She reported a two-month evolving abdominal pain, progressive abdominal distension, and unintentional weight loss. One week before admission, she developed jaundice and progressive dyspnea associated with increased abdominal circumference, prompting hospitalization. Physical examination revealed skin and mucosal jaundice, abdominal distention consistent with ascites, collateral venous circulation development, and abdominal telangiectasias. Laboratory findings revealed normocytic hypochromic anemia (Hb 9.17 mg/dL, MCV 82 fL, MCH 26.1 pg), thrombocytosis (1,688 K/uL), hypoalbuminemia (2.6 g/dL), predominantly direct hyperbilirubinemia (total 4.3 mg/dL, direct 2.7 mg/dL), hyponatremia (124 mmol/L), elevated liver enzymes (AST 358 UI/L, ALT 78 UI/L, ALP 1005 UI/L), and kidney injury (serum creatinine 1.0 mg/dL previously 0.3 mg/dL). A 6-liter evacuative paracentesis yielded ascitic fluid with abnormal cytological characteristics (cellular count 4000 leucocytes/mm3, 93% polymorphonuclear cells, glucose 52 mg/dL, proteins 2800 mg/dL, albumin 1.1 g/dL) and a negative bacterial culture.

**Figure 1:**
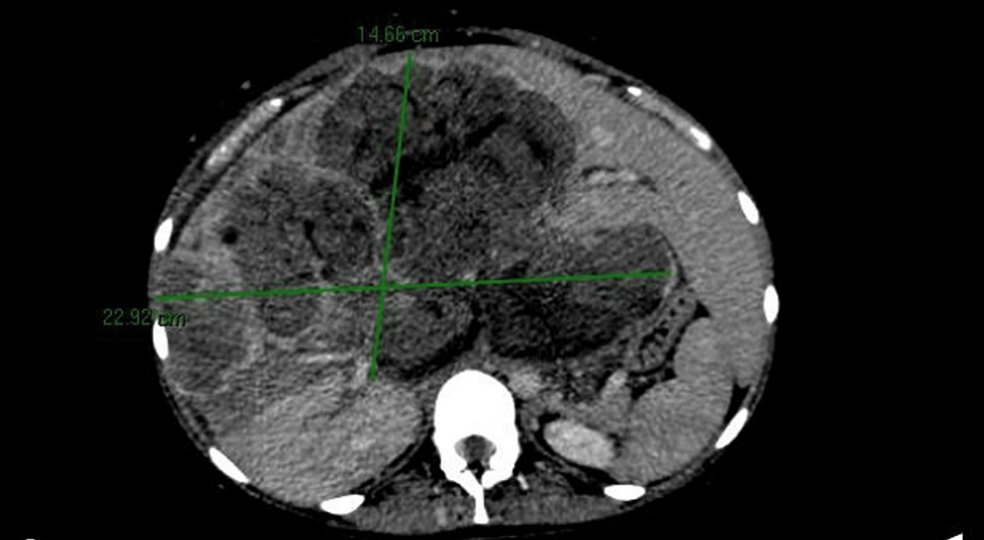
Contrast-enhanced abdomen tomography showing a lobulated hepatic lesion with partially defined borders involving multiple hepatic segments.

A contrast-enhanced abdominal CT scan revealed a lobulated hepatic lesion with partially defined borders involving multiple hepatic segments (Figure 1).

**Figure 2:**
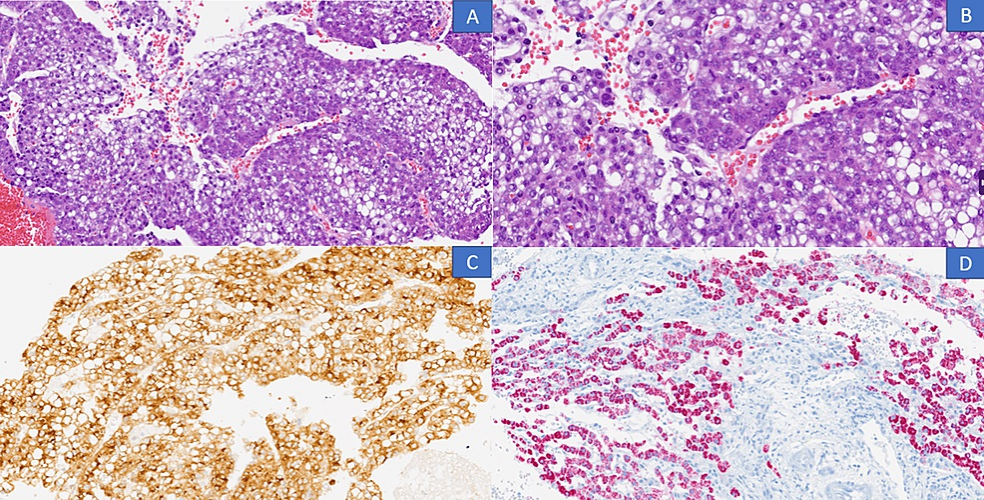
(A, B) Liver biopsy showing fragmented tumor cells arranged in solid sheets with a fetal pattern, pleomorphic alternating with areas of higher differentiation, occasionally organizing around vessels. The cells are large, cohesive, with a small round nucleus and fine chromatin, and indistinct nucleolus; the cytoplasm varies in size, clear or eosinophilic with a fine granular appearance, occasional single-cell apoptosis, and low mitotic activity (hematoxylin and eosin 20x and 40x). (C) Positive Glypican-3 (immunohistochemistry, 20x). (D) Positive HepPar (immunohistochemistry, 20x).

A hepatic biopsy yielded results consistent with an epithelial hepatoblastoma characterized by a predominance of fetal-like cells (Figure 2). 

We consulted the medical oncology service to determine staging and candidacy for oncologic treatment. Utilizing the Pediatric Liver Tumor Staging (PRETEXT) grouping system, we established a stage IV disease considering multifocal characteristics, involvement of the caudate lobe, infiltration into the superior vena cava, portal vein, right and middle hepatic veins, extension to the right thoracic wall, evidence of capsule rupture, ascites, and pulmonary metastases. Following a multidisciplinary discussion, we decided to initiate chemotherapy initiation and strict follow-up.

Cisplatin-based chemotherapy and surgery for children with high-risk hepatoblastoma (SIOPEL-4) treatment protocol was initiated. This protocol involves intravenous chemotherapy with cisplatin and doxorubicin, followed by an assessment of tumor resectability and hepatic transplantation based on clinical response. On the 15th day of chemotherapy, she recurred with jaundice, abdominal pain, leukocytosis, ascites, fever, and acute kidney injury associated with severe hyponatremia. She was readmitted and diagnosed with spontaneous bacterial peritonitis, requiring antibiotic therapy. Furthermore, we noted the development of acute kidney injury in the context of cisplatin-related nephrotoxicity, sepsis, and increased intraabdominal hypertension leading to anuria and an urgent need for renal replacement therapy. She developed altered mental status, somnolence, and persistent renal dysfunction that progressed to multiorgan dysfunction and death.

## Discussion

Hepatoblastoma occurrence after age 5 is uncommon. Most cases are sporadic and not associated with chronic liver disease. In children, it typically presents as an asymptomatic liver mass, but nonspecific symptoms such as abdominal pain, nausea, or vomiting may occur. In more severe cases with significant liver parenchymal involvement, symptoms secondary to portal hypertension may arise, as evidenced in this case [4]. We described the case of a previously healthy 15-year-old adolescent presenting with advanced hepatoblastoma.

Ninety percent of hepatoblastoma cases occur in pediatric patients under five years of age, with a male predominance. It is associated with genetic syndromes and predisposing conditions such as Beckwith-Wiedemann syndrome, familial adenomatous polyposis, and trisomy 18. Late-onset presentation is rare and poorly documented, with only sporadic case reports in the scientific literature. Factors associated with its occurrence include low birth weight, preeclampsia, poly- or oligohydramnios, maternal infertility treatment, parental smoking, and occupational exposure to metals during pregnancy; our patient did not have any of the described risk factors [5]. Regarding its pathogenesis, chromosomal loci 1q, 2q, 4q, 8q, and 20 relate to the loss of heterozygosity and imprinting observed at locus 11p 15.5. Nuclear accumulation of beta-catenin suggests oncogenic alteration of the WNT/beta-catenin pathway, and nuclear accumulation of p53 indicates its involvement in the pathophysiology [6].

In adolescents and adults, the most common symptoms include abdominal pain, abdominal mass, and unintentional weight loss; additionally, symptoms related to liver failure and portal hypertension may be present in the context of cirrhosis, such as ascites and jaundice. In this case, the patient presented with symptoms associated with decompensated chronic liver disease, including ascites, spontaneous bacterial peritonitis, and jaundice. The diagnostic work-up involves imaging studies (abdominal ultrasound, CT abdomen, or MRI), laboratory (alpha-fetoprotein as a clinical and prognostic marker), and the liver biopsy, which can identify various histological subtypes including epithelioid, fetal, mixed, undifferentiated, and mesenchymal hepatoblastoma [7,8]. Imaging findings revealed a lobulated, heterogeneous mass with partially defined borders that enhanced the arterial phase and washed out in the venous phase. The histopathology demonstrated an epithelial pattern with fetal-like cells.

A complete surgical resection represents the cornerstone of treatment. However, effective chemotherapy may affect tumor size and resection adequacy. Treatment decisions are primarily based on the PRETEXT classification, which divides them into four stages, I-IV, depending on the number of hepatic sectors involved. Additionally, annotation factors guide surgical treatment, including hepatic vein involvement, inferior vena cava and portal vein involvement, extrahepatic extension, distant metastases, caudate lobe involvement, multifocal tumor nodules, and tumor capsule rupture [4].

The survival rate has increased in the past decades, especially in the pediatric population, primarily attributed to the introduction of cisplatin-based chemotherapy and orthotopic liver transplantation for locally unresectable diseases. Complete tumor resection is necessary for long-term survival, although there is currently no uniform consensus on the optimal timing. Surgery is the sole option for PRETEXT I and II tumors with clear preoperative venous margins. Neoadjuvant chemotherapy represents the mainstay therapy for patients with unresectable disease. An early referral to a liver transplant center for orthotopic liver transplantation is crucial. Patients with extrahepatic metastases who are not candidates for resection or do not respond to neoadjuvant chemotherapy are not eligible for transplantation. In this case, we decided to initiate the SIOPEL-4 chemotherapy regimen, previously developed by the International Society of Pediatric Oncology (SIOPEL), which includes preoperative chemotherapy with cisplatin and doxorubicin, followed by tumor resection with hepatic transplantation and adjuvant chemotherapy for eligible patients. The prognosis of the disease varies, with survival rates of up to 90% in cases of localized disease with complete surgical resection, whereas it drastically decreases in unresectable or disseminated diseases, as in this case [1,4,9].

Ideally, the management of these rare and complex scenarios involves a multidisciplinary approach, integrating expertise from gastrointestinal, oncology, and liver transplant teams. In this case, our patient presented with a PRETEXT IV hepatoblastoma, rendering her ineligible for initial tumor resection. Consequently, neoadjuvant chemotherapy was initiated. In retrospect, we hypothesize that the acute kidney injury during her initial hospitalization compromised her renal functional reserve. Therefore, the initiation of platinum-based chemotherapy would have increased the risk of further nephrotoxicity. This, combined with immunosuppression leading to spontaneous bacterial peritonitis, exerted significant stress on kidney function, culminating in acute tubular necrosis and necessitating dialytic support. Ultimately, this cascade of events led to multiorgan failure and death. Although there are recommendations for preventing and managing cisplatin-induced nephrotoxicity, such as infusion of intravenous fluids with chemotherapy and monitoring of kidney function, our patient developed a rapid decline in kidney function, limiting the possibility of performing early therapeutic intervention.

## Conclusions

This report describes the case of a previously healthy female adolescent diagnosed intrahospital with stage IV epithelial hepatoblastoma. The SIOPEL-4 chemotherapy resulted in an unfavorable course, ultimately leading to the patient's death due to complications. Although rare beyond the age of five, adult/adolescent hepatoblastoma may occur when encountering hepatic masses suggestive of neoplasm and no inherent risk factor history for cirrhosis or hepatocarcinoma. Clinical physicians need to consider this neoplasm as a differential diagnosis. Further investigation, case reporting, and literature case reviewing may complement the existing literature and standardize hepatoblastoma treatment in older people.

## References

[1] Pateva IB, Egler RA, Stearns DS (2017). Hepatoblastoma in an 11-year-old: case report and a review of the literature. Medicine (Baltimore).

[2] Zhong S, Zhao Y, Fan C (2018). Hepatoblastoma with pure fetal epithelial differentiation in a 10-year-old boy: a rare case report and review of the literature. Medicine (Baltimore).

[3] Al-Jiffry BO (2019). Adult hepatoblastoma: an update. Egypt J Hosp Med.

[4] Czauderna P, Garnier H (2018). Hepatoblastoma: current understanding, recent advances, and controversies. F1000Res.

[5] Kahla JA, Siegel DA, Dai S, Lupo PJ, Foster JH, Scheurer ME, Heczey AA (2022). Incidence and 5-year survival of children and adolescents with hepatoblastoma in the United States. Pediatr Blood Cancer.

[6] Wang YX, Liu H (2012). Adult hepatoblastoma: systemic review of the English literature. Dig Surg.

[7] de Bree K, Westermann AM, Klümpen HJ, Verheij J, Phoa SS, Oomen M, van Gulik TM (2018). Two cases of hepatoblastoma in young adults. J Adolesc Young Adult Oncol.

[8] Celotti A, D'Amico G, Ceresoli M, Tomasoni M, Raimondo S, Baggi P, Baiocchi GL (2016). Hepatoblastoma of the adult: a systematic review of the literature. Surg Oncol.

[9] Al-Jiffry BO (2013). Adult hepatoblastoma: a case report and literature review. Int J Surg Case Rep.

